# Supplement of Betaine into Embryo Culture Medium Can Rescue Injury Effect of Ethanol on Mouse Embryo Development

**DOI:** 10.1038/s41598-018-20175-w

**Published:** 2018-01-29

**Authors:** Di Zhang, Huaijiang Jing, Changfeng Dou, Ling Zhang, Xiaoqing Wu, Qingqing Wu, Haoyang Song, Dengkun Li, Fengrui Wu, Yong Liu, Wenyong Li, Rong Wang

**Affiliations:** 1 School of Biological and Food Engineering, Fuyang Teachers College, Fuyang, 236037 China; 2 Key Laboratory of Embryo Development and Reproductive Regulation in Anhui, Fuyang, 236037 China

## Abstract

Mammal embryos can be impaired by mother’s excessive ethanol uptake, which induces a higher level of reactive oxygen species (ROS) and interferes in one carbon unit metabolism. Here, our analysis by *in vitro* culture system reveals immediate effect of ethanol in medium on mouse embryo development presents concentration dependent. A preimplantation embryo culture using medium contained 1% ethanol could impact greatly early embryos development, and harmful effect of ethanol on preimplantation embryos would last during the whole development period including of reducing ratio of blastocyst formation and implantation, and deteriorating postimplantation development. Supplement of 50 μg/ml betaine into culture medium can effectively reduce the level of ROS caused by ethanol in embryo cells and rescue embryo development at each stage damaged by ethanol, but supplement of glycine can’t rescue embryo development as does betaine. Results of 5-methylcytosine immunodetection indicate that supplement of betaine into medium can reduce the rising global level of genome DNA methylation in blastocyst cells caused by 1% ethanol, but glycine can’t play the same impact. The current findings demonstrate that betaine can effectively rescue development of embryos harmed by ethanol, and possibly by restoring global level of genome DNA methylation in blastocysts.

## Introduction

High concentrations of ethanol can impact physiological status of somatic cell and germ cell^1–3^, especially excess alcohol intake would badly harm various tissue and organs, such as liver and heart, and make cancer^4–6^. It is hard to clarify how ethanol to interfere in cell action for ethanol leading a serial change in physiology, signal transduction and gene expression^7–9^. Even more, ethanol being a teratogen at higher concentration can cause a serial of embryo abnormal changes and defects referred to fetal alcohol spectrum disorders (FASD)^10^. In some cases, ethanol was used as one of stimulation methods in the study of mouse egg activation^11^ and as a cryoprotectant in embryo cryopreservation^12^. More often, ethanol was used to solve the drug added into embryos culture medium.

In all studies, the adverse influences of ethanol to fetuses attract attention in that it had been well known that excessive alcohol intake could induce a variety of fetal malformation, which would bring a longer suffering life to recipient. Moreover, negative impact of ethanol on physical health can be transferred to offspring through influencing germ cell or embryo development. Data indicate that the mouse early embryos suffered acute doses of alcohol is susceptible to the deleterious effects of fetal death and intrauterine growth retardation^13^. Ingestion of 10% ethanol for 15 days can cause a significantly reduction in the ratio of blastocyst hatching, and progestational intake of alcohol might impair trophoblast invasion^14^. Studies from *in vitro* culture show that ethanol might exert a toxic impact to porcine embryo development just at low concentrations in that treating oocytes with ethanol over 1% during *in vitro* mature (IVM) can raise the level of ROS in cells and upregulate apopotosis related gene expression^15^. But study of precise dosage effect of ethanol on mouse embryo development has not yet been investigated very well so far.

Ethanol induced ROS can destroy the structure and properties of protein, and then make the change of enzyme activity^16^. But it is hardly to elucidate the pathway and molecular mechanism, underlying which how ethanol impact embryo development. Of all metabolism processes interfered by ethanol, one-carbon metabolism involved folate as a methyl donor is influenced strikingly^17^. One carbon unit can be derived from various dietary, and abnormal one carbon metabolism could impact process of DNA methylation. Existed data prove that ethanol can interfere with folate-dependent biochemical reactions and inhibit folate-mediated synthesis to interrupt methylation process mediated by S-adenosylmethionine (SAM)^4,18^. In mouse, study indicates that the exposure of ethanol to preimplantation embryo can change the level of DNA methylation in the imprinting control region (ICR) of H19^19^. Based on the findings of the epigenetic effect of ethanol on genome DNA methylation and of interferences to one carbon metabolism, further investigation reveals that supplement of floate is helpful to modulate DNA methylation and reduce potential cancer risk by using animal model or *in vitro* culture^20^. Indeed, supplement of folate could block ethanol-induced fetal teratogenesis, and effectively block neural tube defects and congenital anomalies caused by ethanol in both human body and animal experiment^21^. But usage of folate in human body is restricted because the range of folate toxicity to cellular and human physiological functions remains unclear.

As being another methyl donor in the cycle of one carbon metabolism, betaine is widely distributed in animals, plants and microorganisms. It has been well known that betaine can also protect liver against ROS induced by ethanol, and supplement of betaine can prevent ethanol induced rising levels of MDA, GSH, CYP2E1 and ALT in liver^22,23^. The source of betaine in early embryos has not been fully known, but studies suggest that preimplantation embryos contain nearly constant levels of endogenous betaine till morula stage, and partly endogenous betaine in oocytes possibly come from cumulus cells during meiotic maturation, and SIT1 as being one of betaine/proline transporters is activated in mouse eggs after fertilization and play a key role for betaine being transported into oocyte until the 2-cell stage^24,25^. Furthermore, betaine might play protective role through holding normal osmolarity of cell^26^, and addition of betaine into culture medium can also protect embryo against change of osmolarity^27,28^. However, it remains unknown that how betaine immediately exerts the impact on the development of ethanol exposed embryos as so far.

To investigate specificity and possible route of betaine to rescue development of early embryos exposed to ethanol, in this present study, we first examined that the impact of gradient concentration of ethanol on development of mouse embryo, then analyzed the efficiency of adding betaine into culture medium to improve performance of embryo development. Besides these, this study also investigated the status change of global level of genome DNA methylation of blastocyst resulted from different group. Our results show that supplement of betaine into medium can effectively improve development of ethanol treated mouse embryos including blastocyst formation, implantation, and postimplantation development, and recover the level of global DNA methylation in ethanol treated blastocyst to normal status. All these suggest that further investigation is needed to reveal the possibility of betaine being an agent used in reproduction therapy.

## Results

### Effect of ethanol on Mouse preimplantation embryo development depends on its concentration in culture medium

**Figure 1:**
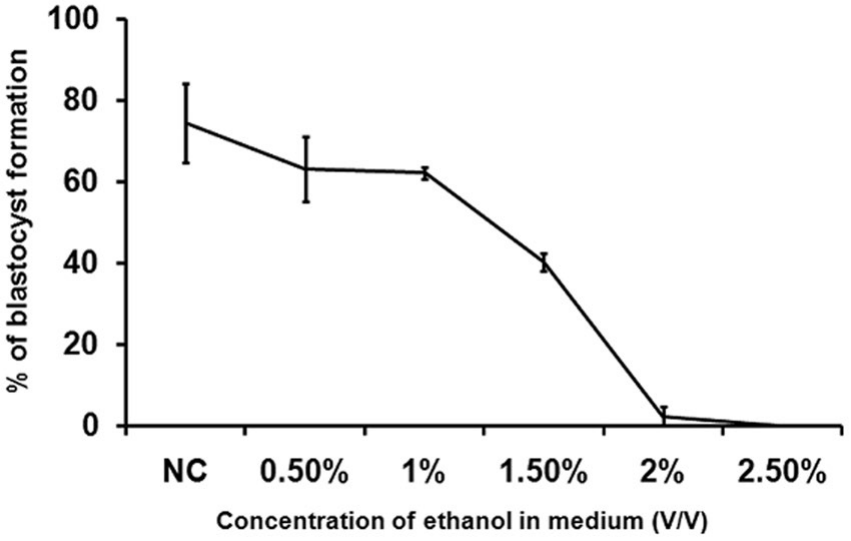
Effect of ethanol at different concentration on preimplantation embryo development. Pronuclear embryos (PN), obtained from *in vitro* fertilization (IVF) after sperm being added into medium contained oocytes about 8 hours, were respectively cultured in KSOM medium contained different concentration of ethanol for 4 days. Blastocysts formation was analyzed by percentage of number of blastocyst to number of pronuclear embryos used in each treatment group. Data showed ethanol might obviously reduce ratio of blastocysts formation when its concentration was 0.5% and above. The data shown are means ± SEM.

To investigate the immediately effect of ethanol on embryo development at preimplantation stage, and the relationship between this effect and concentration of ethanol in medium, we cultured pronuclear (PN) embryos derived from IVF eggs in culture medium containing ethanol, and then examined their developmental profile of blastocyst formation. Results in Table 1 show that when the concentration of ethanol in medium rises to 0.5% and above, ethanol appears the ability to inhibit the early development of mouse embryos at each stage. At Day 5, all embryos were blocked at 4-cell stage when the concentration of ethanol in medium reaches 2.5%. The percentage of blastocyst formation would significantly reduce to 40.3% when ethanol concentration reach 1.5%, and almost all embryos were inhibited when ethanol concentration rise to 2.0% (Fig. 1). These data appear to be some degree of similarity with previous finding^29^, and this study also indicates that inhibitory impact of ethanol on blastocyst formation takes on concentration depended, although effect of 0.5% and 1% ethanol seems to promote development a little from 2-cell stage to morula stage although ratio of their blastocyst formation obviously reduces.

**Table 1:** Effect of ethanol at different concentrations on mouse preimplantation embryo development^a^.

Ethanol Concn. % (V/V)	No. of zygote used (N)	Development stage				
		2-cell (%)	4-cell (%)	8-cell (%)	Morula (%)	Blastocyst (%)
0	94	89.60 ± 8.31	86.31 ± 9.92	80.60 ± 8.02	77.38.38 ± 9.92	74.44 ± 9.62
0.5	60	93.10 ± 6.70	85.04 ± 1.17	81.59 ± 2.23	73.08 ± 7.56	63.07 ± 7.90
1.0	221	95.51 ± 1.00	77.65 ± 6.91	74.53 ± 17.07	70.72 ± 3.07	62.21 ± 4.65
1.5	118	93.94 ± 3.03	88.23 ± 5.78	83.96 ± 5.30	75.18 ± 9.82	40.30 ± 2.14
2.0	122	88.12 ± 7.69	26.31 ± 13.03	15.68 ± 15.68	15.68 ± 15.68	2.35 ± 2.35
2.5	48	68.18 ± 25.71	1.92 ± 2.72	0	0	0

^a^Data are presented as means ± SD.

Besides of above tests, PN embryos were also cultured in medium containing 0.1% ethanol for 4 days (data no present here), and no significant difference can be found between control group and test group, even slightly better for blastocyst formation of test group than of control group. Previous study shows that normal development of mouse embryos can be blocked on 2-cell stage when culture medium contains ethanol at concentration of 10%, but in this study, all embryos stop to develop at 2-cell stage when concentration of ethanol in medium rise to 2.5%. Thus, in the followed work, we try to clarify whether or not supplement of betaine into medium is helpful to improve the impaired effect of ethanol on mouse embryo and underlying mechanism by embryos culture with medium contained 1% or 1.5% ethanol.

### Betaine could reverse the impact of ethanol on preimplantation embryo development

**Figure 2:**
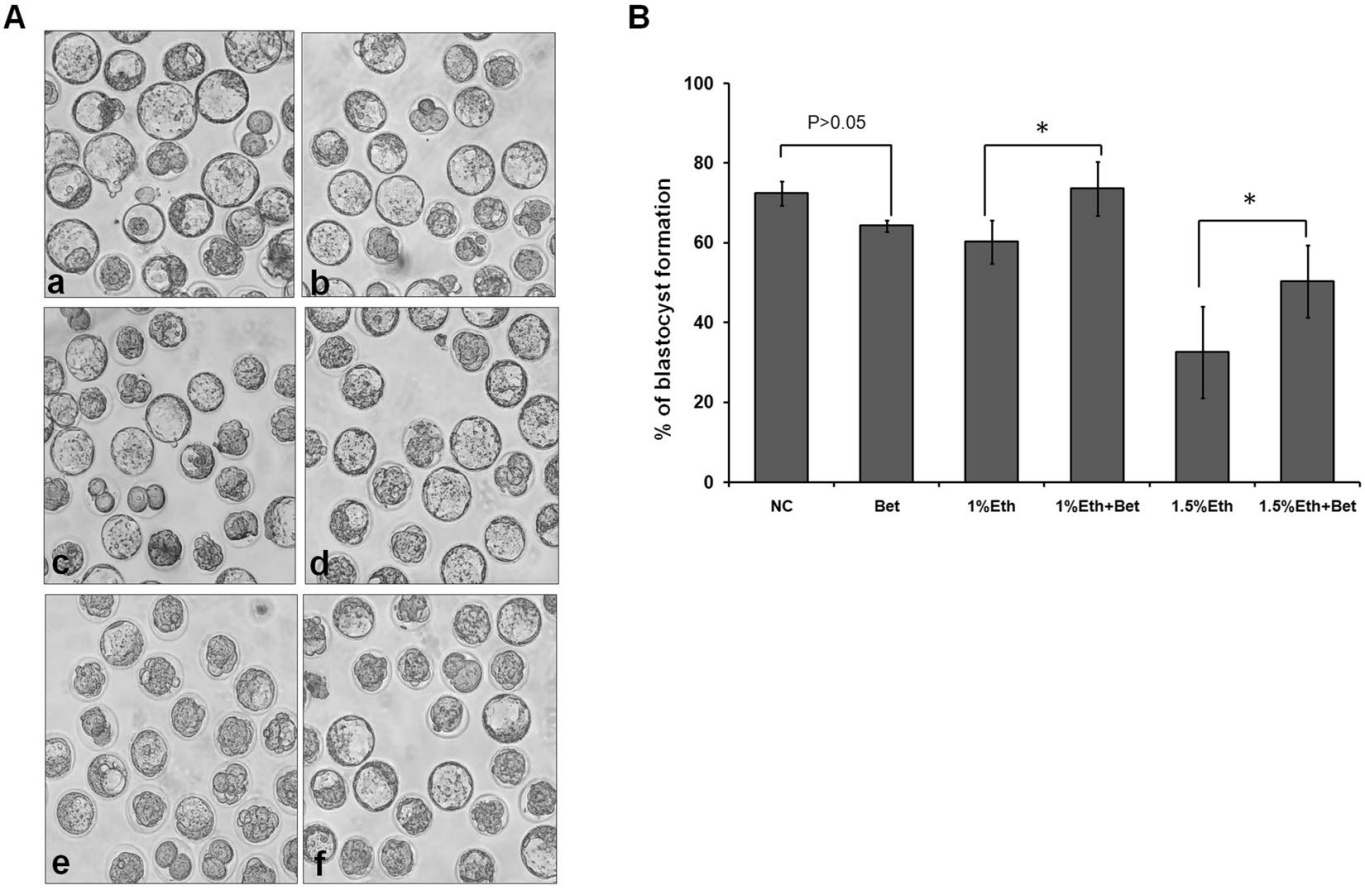
Effect of ethanol and betaine on blastocyst formation. Pronuclear embryos (PN), obtained from *in vitro* fertilization (IVF) after sperm added into medium contained oocytes about 8 hours, were respectively cultured in KSOM medium supplemented with ethanol or (and) betaine for 4 days. Developmental profile of embryos was analyzed. (**A**) Morphology of blastocyst resulted from different culture medium. Within (**A**), a referred to control group (NC), no ethanol or betaine in its medium, c and e referred to Eth group, whose medium contained ethanol of concentration 1% and 1.5% respectively, b,d and f referrred to supplement of betaine 50 μg/ml into medium contained ethanol corresponding to a, c and e. (**B**) Effect of ethanol and betaine on ratio of blastocyst formation. Data showed supplement of betaine into medium can effectively restore blastocyst formation blocked by ethanol of concentration 1% or 1.5%. The data shown are means ± SEM. Values are statistically different (p < 0.05,*).

At the beginning of examining the reversal impact of betaine, we tested the effect of various concentration of betaine in medium on mouse embryo development, and searched related work about dosage of betaine impacting on animal cell. Results indicate the ratio of blastocyst formation don’t change obviously under condition of sole adding 50 μg/ml betaine into medium (data no shown here). Subsequently, we analysed impact of different concentrations of betaine on embryo development. Results in Fig. 2B showed that supplement of 50 μg/ml betaine into medium could effectively improve development of embryos cultured in medium containing 1% or 1.5% ethanol for an over 10% raised ratio of blastocyst formation (p < 0.05)(Table 2). Then, the morphology of early embryo didn’t present difference among all test groups (Fig. 2A). Thus, it might let to believe that betaine is an effective reagent to protect embryo against impairing by ethanol.

**Table 2:** Effect of betaine on ethanol-treated embryos for blastocyst formation.

Group	Ethanol Concn. (V/V)%	Betaine (50 μg/ml)	No. of zygote used (N)	Ratio of blastocyst formation (%)^a^
NC	0	−	402	72.44 ± 2.97
Bet	0	+	128	64.29 ± 1.42
1%Eth	1.0	−	529	60.25 ± 5.41
1%Eth + Bet	1.0	+	523	73.65 ± 6.72
1.5%Eth	1.5	−	247	32.54 ± 11.53
1.5%Eth + Bet	1.5	+	161	50.40 ± 9.04

^a^Data are presented as means ± SD.

### Betaine could alleviate the level of ROS in early embryo cells and reverse impact of ethanol on preimplantation embryo development

**Figure 3:**
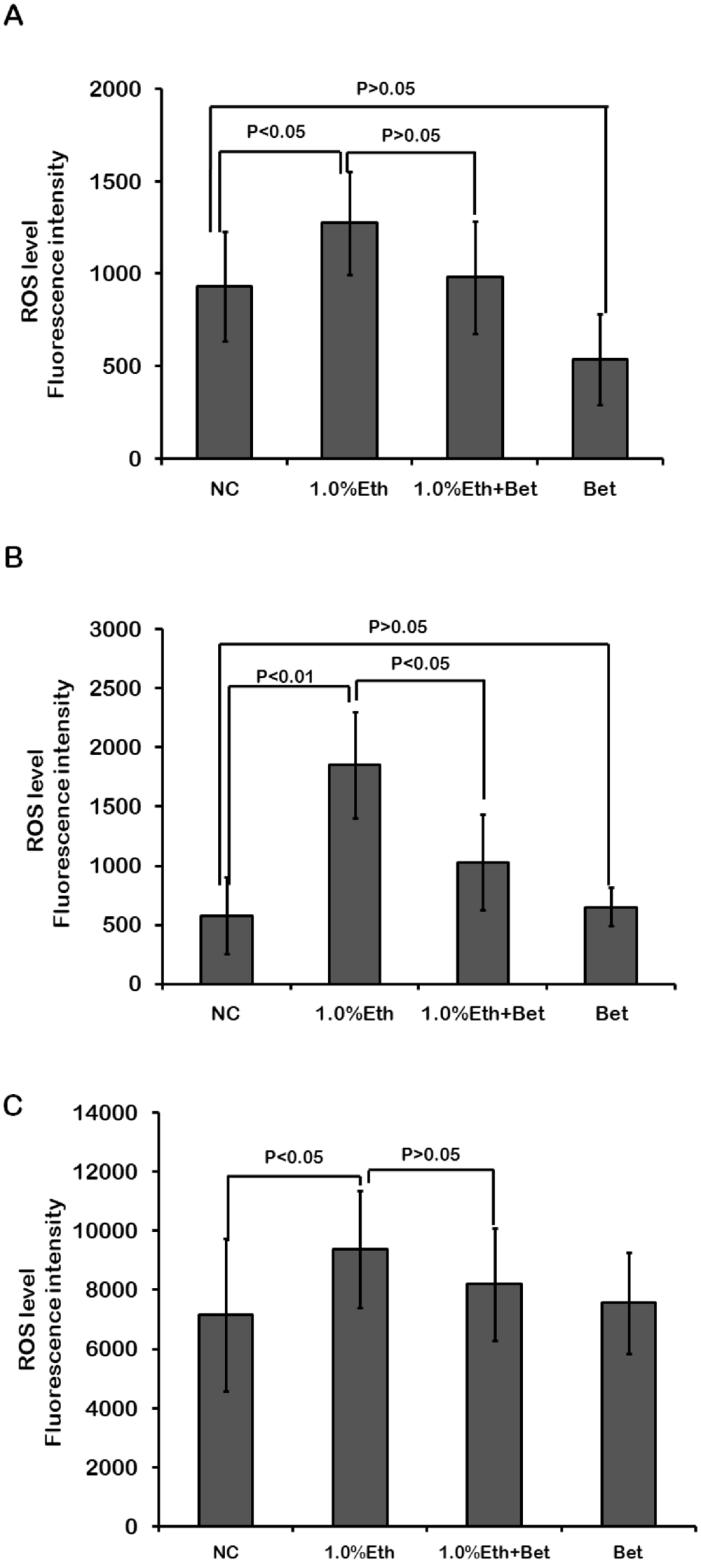
Effect of ethanol and betaine on the level of ROS in cells of early mouse embryos. The change of the level of ROS in embryo cells cultured medium with 1.0% ethanol (1.0% Eth group), betaine (Bet group) and 1.0% ethanol plus betaine (1.0% Eth + Bet group) at PN stage, and the same test on the level of ROS is carried out at 2-cell stage (**B**) and morula stage (**C**). In each test, the level of ROS appears to be higher obviously in 1.0% Eth group than in NC group. Addition of betaine into medium can reduce the level of ROS in all three tests, especially in 2-cell embryos (p < 0.05).

It remains unclear that if or not betaine could directly protect embryos against damage from their environment. By monitoring the fluorescence intensity of embryo cells at three different stages, effect of betaine on ethanol raised level of ROS can be analyzed. In Fig. 3, results show that ethanol can cause an obviously increased level of ROS in embryo cells of 1% ethanol treatment group (Eth) compared to control group (NC) at both 1-cell stage (p < 0.05), 2-cell stage (p < 0.01) and morula stage (p < 0.05). Data also indicate that the level of ROS significantly decrease in embryo cells of 1% Eth + Bet group, compared to 1% Eth at 2-cell stage (p < 0.05). Meanwhile, the of level of ROS show a trend of slightly downward in embryo cells of 1% Eth + Bet group, compared to 1% Eth at 1-cell or morula stage despite of the difference is not significantly. Thus, our experiments verify the speculation that betaine could really alleviate injury effect of ethanol on embryo especially at early stage of preimplantation embryos.

### Supplement of betaine in medium can restore the performance of blastocyst implantation and post-implantation development inhibited by ethanol

**Figure 4:**
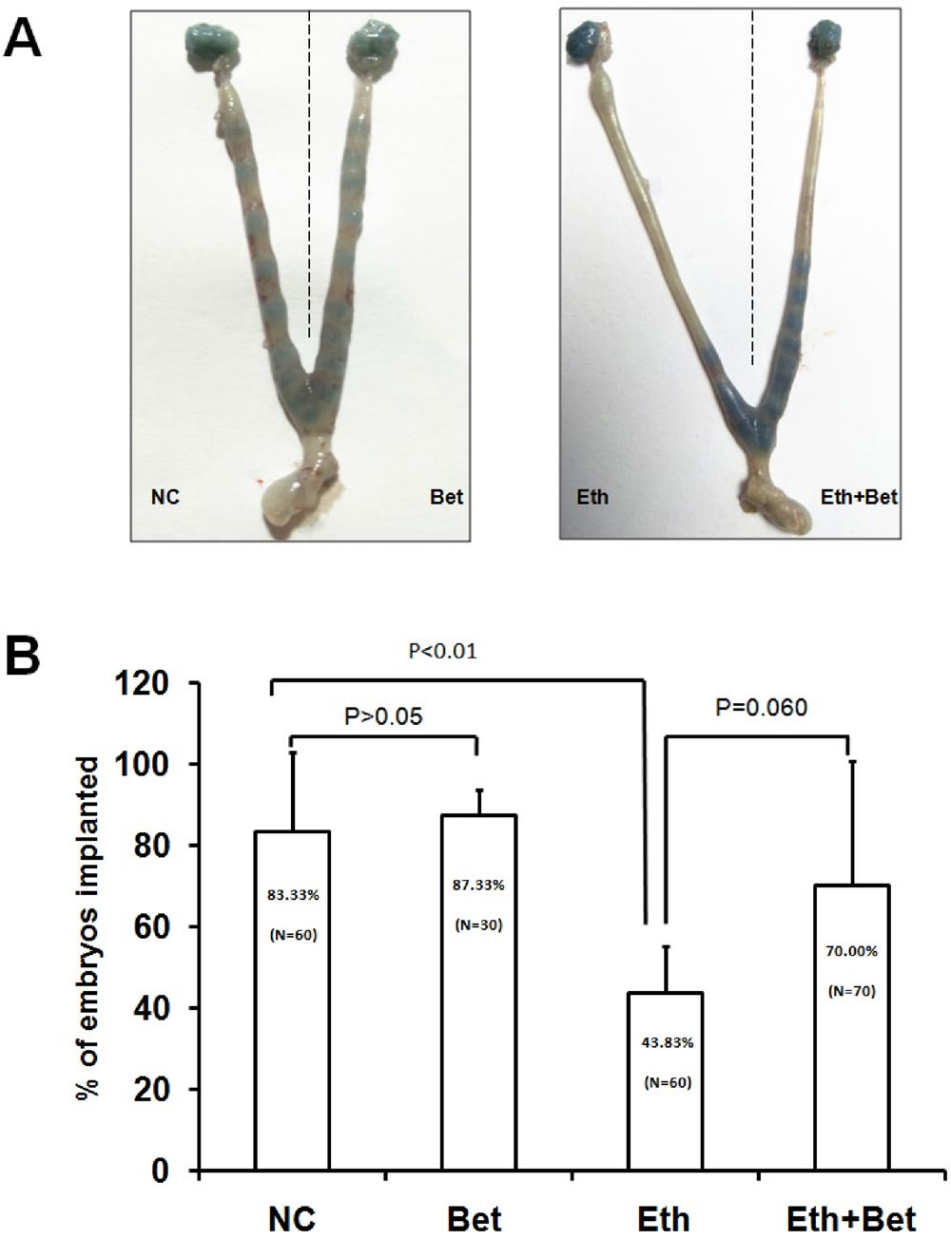
Effect of ethanol and betaine on blastocyst implantation. Transferred Blastocysts were obtained from each treatment group including control group (NC) without both ethanol and betaine in embryo culture medium, medium of betaine group (Bet) contained betaine (50 μg/ml), medium of ethanol group (Eth) contained ethanol (v/v 1%), and medium of betaine + ethanol group (Eth + Bet) contained betaine (50 μg/ml) and ethanol (v/v 1%). In experiments, ten blastocysts were transferred into each uterine horn of the recipient. After transferring 24 and 30 h, Implantation sites were examined by an intravenous injection of 0.1 ml of 1% Chicago blue B for 5 min as described in Paria’s work (Paria BC *et al*., 1993). (**A**) Effect of supplement of ethanol or (and) betaine into culture medium on blastocyst implantation. The performance of Implantation sites was checked by injection of 100 μl of 1% Chicago blue dye solution 24 hours after blastocysts being transferred into horn of uterus. (**B**) Quantitation of results. The numbers within parentheses indicate the percentage of blue bands to blastocyst transferred and total number of blastocyst used in each group. Values are statistically different, p < 0.05.

To survey if ethanol could impair blastocyst implantation or not and betaine repairs this harm effect, we analyzed the proportion of embryo implantation by transferring differently treated blastocyst into uterus of pseudopregnant female mouse. Success of blastocyst implantation can be signed by counting number of implantation sites which are stained with injection Chicago blue dye 24 hours after embryo transferring. Result as shown in Fig. 4, the proportion of implanted sites in recipient uterus shows a non-significantly difference between NC group (mean = 83.33%) and Bet group (mean = 87.33%). The implantation rate of blastocysts submitted to 1% ethanol treatment of Eth group (mean = 43.83%) was significantly lower than that of NC group (mean = 83.33%, P < 0.01). Despite of non-significantly difference presented between implantation ratio of Eth group and of Eth + Bet group (P = 0.06), but the percent of blastocysts implantation rise great to 70.00%.

**Figure 5:**
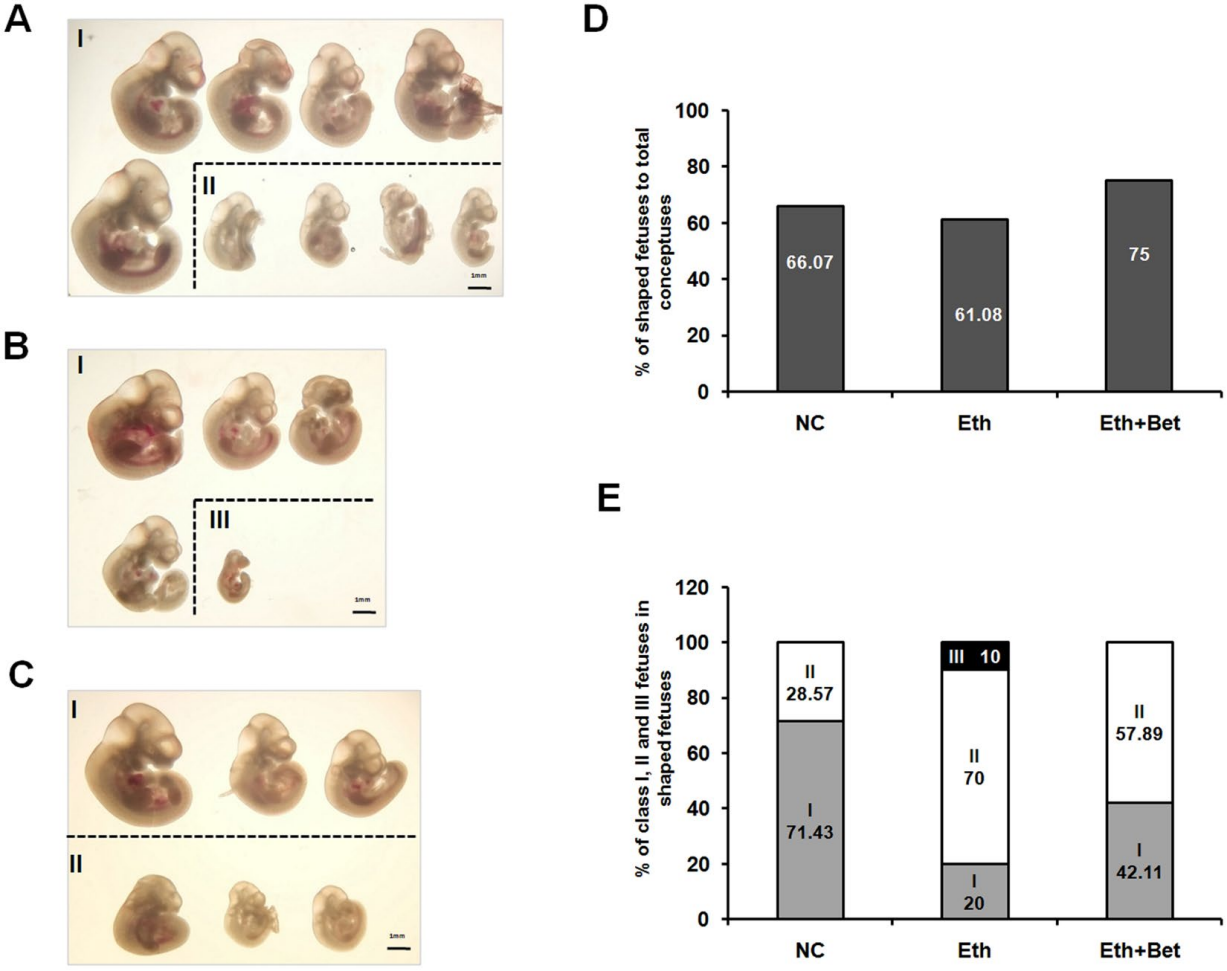
Effect of ethanol and betaine on the development of embryos (fetuses) after blastocysts transferring 8 days (E11.5). (**A**) Fetuses of control group (NC) from transferred IVF blastocysts cultured in KSOM medium without any supplement for 4 days. They show typical morphological development with some degree of size difference, and are recorded in class I or class II according to their size. The scale is given by a small bar (10 mm). (**B**) Fetuses of ethanol treatment group (Eth) from transferred IVF blastocysts cultured in KSOM medium contained 1% ethanol for 4 days. They also show varying morphological development with even largely difference in size, and these smaller fetuses are recorded in class III according to their size and shape. (**C**) Fetuses of ethanol plus betaine treatment group (Eth + Bet) from transferred IVF blastocysts cultured in KSOM medium contained 1% ethanol and 50 μg/ml betaine for 4 days. Morphology quality of these fetuses present alikely to of NC group. (**D**) and (**E**) Quantitative analysis of ethanol and betaine affecting postimplantation embryos development. Results in (**D**) indicate that percentage of shaped fetuses in all conceptuses could be restore to a higher level in Eth + Bet group than in Eth group (75% vs 61.08%, P > 0.05), even than in NC group (75% vs 66.07%, P > 0.05). These suggest betaine might be an effective regent to protect embryos development. Alike, it can be found in (**E**) that betaine could rise the percentage of class I fetuses to 42.11% in Eth + Bet group, while, only 20% of class I fetuses in shaped conceptuses of Eth group, and calss III fetuses don’t appear in Eth + Bet group despite values are not statistically different.

To assess impact of ethanol on long term embryo development, and if supplement of betaine into medium can effectively restore normal postimplantation development, blastocysts from different group including 1% ethanol (Eth), 1% ethanol + 50 μg/ml betaine (Eth + Bet) and control group (NC) were transferred into uterus. After 8 days, the mice were killed to collect the uteruses to obtain fetuses. As shown in the Fig. 5D, at day 8 after embryo transferring, by collecting conceptuses and obtain shaped fetuses, it was found that the ratio of shaped fetuses conceptuses in all conceptuses decreased to 61.08% in 1% ethanol treatment group (Eth), but rose to 75% in Eth + Bet group despite statistical analysis did not show a significant difference between them (P > 0.05). According to the morphology aspect and size of fetuses obtained, all shaped fetuses in three treatment group could be divided into three types, namely calss I, II and III (in Fig. 5A,B and C). Class I fetuses presented a normal size and morphological aspect. Class II fetuses presented some degree of little size but morphological aspect. Class III fetuses presented remarkably little size and developmental lagging. Results in Fig. 5E show that supplement of 50 μg/ml betaine into medium is capable of improving development of fetuses from ethanol treated blastocysts. The proportion of classes I fetuses in 1% ethanol treatment group (Eth) reduced to 20%, but in the other treatment, the mean ratio of classes I fetuses were, respectively, 71.43% (P > 0.05) in control group (NC), 42.11% (P > 0.05) in 1% ethanol + 50 μg/ml betaine group (Eth + Bet). Class III fetuses only existed in Eth group (10%). Thus, it could be confirmed solidly that betaine owned the ability of reversing harm impact of ethanol to mouse embryos. All these results indicate that supplement of betaine in medium can effectively enhance postimplantation development of embryos impaired by ethanol. So we analyzed the possible potential causes in the subsequent experiments.

### Glycine couldn’t rescue embryo development impaired by ethanol

**Figure 6:**
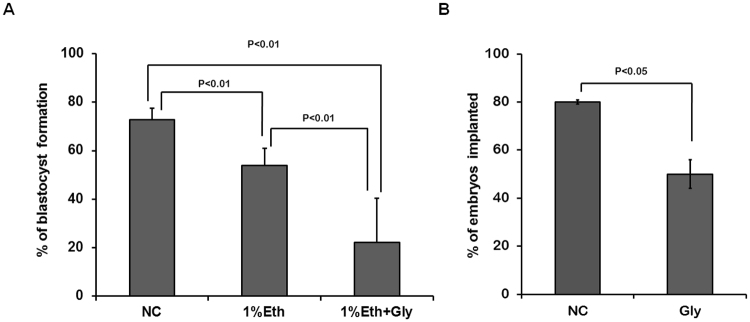
Ethanol damaged embryo development can not be rescued by Glycine. (**A**) Glycine further deteriorates formation of blastocyst cultured in medium with 1% ethanol. Pronuclear embryos (PN), obtained from *in vitro* fertilization (IVF) after sperm added into medium contained oocytes about 8 hours, were respectively cultured in KSOM medium without (NC) or with contained 1% ethanol (Eth), 1% ethanol plus 23 μg/ml glycine (Eth + Gly) for 4 days. Developmental profile of embryos was analyzed. Data showed glycine could not restore ethanol-reduced blastocyst formation, even obviously caused a lower ratio of blastocyst formation (to 22.2%). The data shown are means ± SEM. Values are statistically significantly different (p < 0.01, **). (**B**) Effect of glycine on blastocyst implantation. Ten blastocysts cultured in medium with glycine (32 μg/ml) or without are transferred into horn of one uterus of the same mouse. After transferring 24 h, implantation sites were examined as described before. Data show percentage of blastocyst obviously decrease under existence of glycine in culture medium, values are statistically different, p < 0.05. All these indicate that glycine is unable to take the place of betaine to restore ethanol impaired embryo development.

One interesting question is if betaine could improve early embryo development under condition of medium contained ethanol through its regulating osmotic stress of embryo cell. To answer that, an experiment was designed to investigate whether or not glycine, an analogue of betaine without methyls, could play the same role to rescue the development of ethanol impaired embryos because glycine was often used to learn mechanism of osmotic pressure and oxidative damage in previous studies^30,31^. We carried out a test with supplement of glycine into medium contained 1% ethanol to culture embryos instead of betaine. Here, the concentration of glycine was adopted at 32 μg/ml in medium for the same molar concentration as betaine at 50 μg/ml used in this study. Results show (Fig. 6A) that glycine can not restore ethanol impaired mouse embryo development, but even obviously reduced ratio of blastocyst formation (p < 0.01). Besides that, in control test, sole supplement of glycine into medium would present negative impact when its concentration was over 10 μg/ml (data no present here). On the contrary, when concentration of betaine in medium reach 500 μg/ml, its inhibitory impact on embryo development just appear slightly, which indicates a widely concentration range of betaine could be adopted safely.

To further find out the impact of glycine on embryo implantation and post-implantation development, ten blastocysts cultured in medium with glycine (32 μg/ml) or without are separately transferred into horn of one side uterus of the same mouse. As shown in the below (Fig. 6B), the proportion of implanted sites in recipient uterus shows a significantly difference between Negative control group (NC, mean = 80.00%) and Glycine group (Gly, mean = 50.00%). In the aspect of post-implantation embryo development, fifty blastocysts of NC group or Gly group used to be transferred into uterus as described in method. Morphology performance of embryos (E11.5) was analyzed by collecting shaped fetuses in conceptuses of NC group or Gly group after blastocyst transferring 8 days. Average ratio of shaped fetuses is 68% in NC group (n = 34) and 46% in Gly group (n = 23) (p = 0.059). Besides, obviously distinction is found between two treatment groups for the number of fetuses belonging class III in Gly group (n = 4 in 23) is higher than in NC group (n = 1 in 34) despite probability is not significantly. So, we might draw the conclusion that glycine can cause a inverse impact on embryo development when glycine supplemented into embryo culture medium at the same molar concentration as betaine does. All these results hint us that betaine does play a peculiar effect on embryo development including on embryo implantation process, and the protection of osmotic pressure in embryo cell is not main cause to rescue embryo development impaired by ethanol in that normal embryo development could be blocked by glycine here.

### Betaine can raise the global level of 5-MeC of genome DNA in blastocysts cultured in medium contained 1% ethanol

The developmental status of embryos at blastocyst stage is pivotal to the destiny of post-implantation embryo development for programmed gene expression need properly be carried out through establishment of epigenetic modification in genome DNA and histone level. Now, it had been learned that DNA methylation play a key role in regulation of gene expression during embryos development as called genomic imprinting. More importantly, it is a key stage that the global DNA methylation is reestablished at blastocyst stage during embryo development, and difference level of DNA methylation will present in lineage of inner cell mass (ICM) and trophectoderm (TE) respectively. To determine if betaine could rescue properly establishment of DNA methylation damaged possibly by ethanol, we checked the global level of genome DNA methylation in blastocysts from different treatment groups by immunofluorescence with anti 5-methylcytosine (5-MeC) antibody.

**Figure 7:**
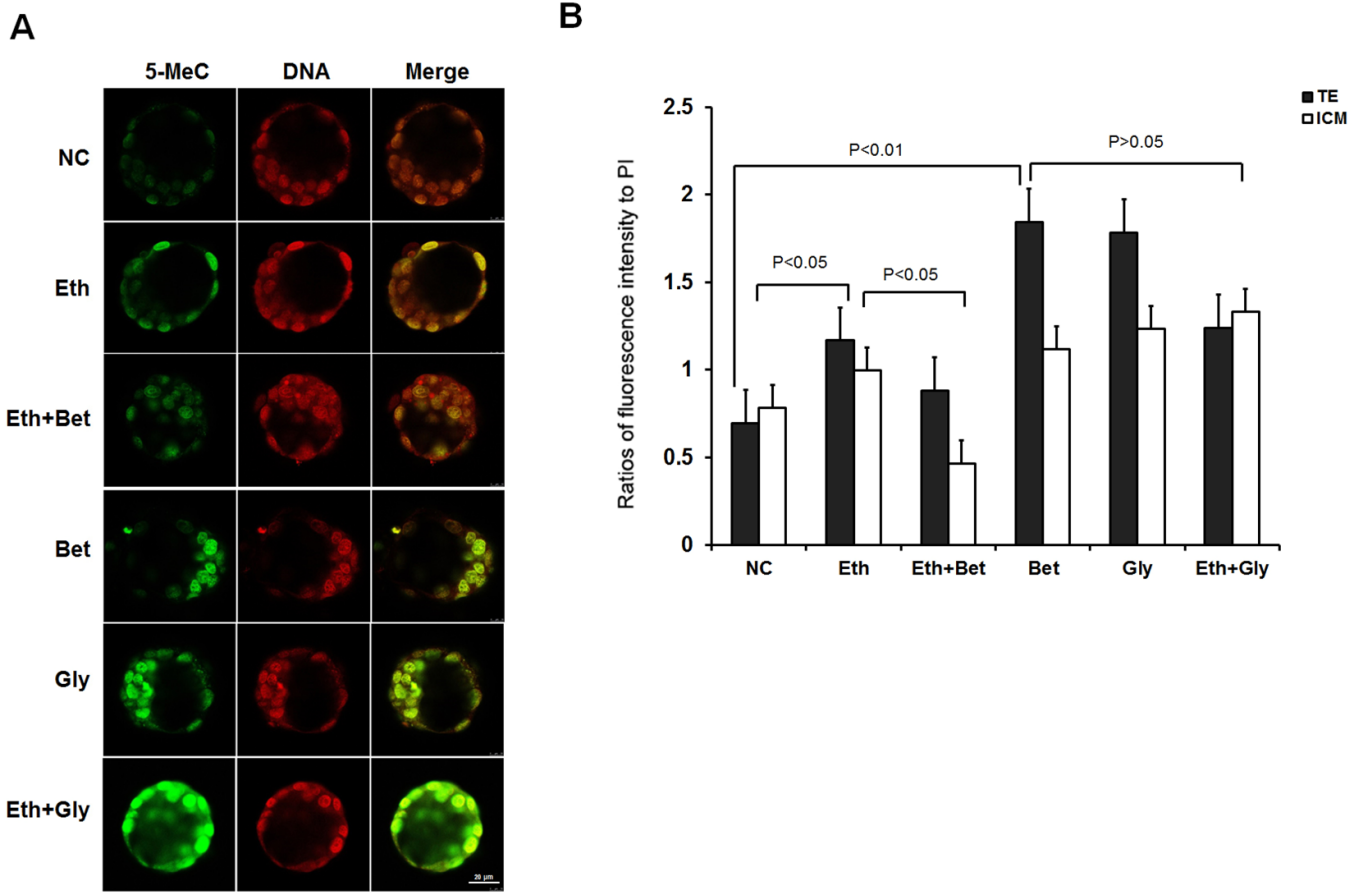
Analysis of global level of DNA methylation in blastocysts from culture medium contained ethanol, betaine or glycine. (**A**) Representative images of 5-MeC immunofluorescence in blastocysts from control group (NC), ethanol treatment group (Eth) whose medium contained 1% ethanol, ethanol plus betaine treatment group (Eth + Bet) whose medium contained 1% ethanol and 50 μg/ml betaine, betaine treatment group (Bet) whose medium contained 50 μg/ml betaine, glycine treatment group (Gly) whose medium contained 23 μg/ml glycine, and ethanol plus glycine treatment group (Eth + Gly) whose medium 1% ethanol and 23 μg/ml glycine (Eth + Gly) for 4 days. The blastocysts were stained with 5-MeC specific antibody (green) and counterstained with PI (red). (**B**) Quantitative analysis of global levels of 5-methylcytosine (5-MeC). Average ratios of fluorescence intensity were obtained by dividing the intensity of the specific target (5- MeC) by the intensity of DNA stain (PI). A one-way ANOVA model used to examine differences between treatment groups. Values are statistically different, p < 0.05.

Results in Fig. 7 show that both of sole supplement of ethanol (1%) and betaine (50 μg/ml) might cause an obvious rising of global level of DNA methylation in ICM and TE linage of blastocyst, even a more higher level in betaine group. However, an obvious decreasing of the DNA methylation level in ICM and TE linage of blastocyst can be found in Eth + Bet group (mean = 0.88, 0.47, P < 0.05). Likely, glycine also greatly increases the level of DNA methylation when it being added into medium without ethanol by the same molar concentration of 50 μg/ml betaine (0.427 mM) used in this study (glycine, 32 μg/ml equated to 0.427 mM), but it was unable to reduce the level of global DNA methylation in ethanol treated blastocysts (P > 0.05).

## Discussion

The present study focuses on the direct impact of ethanol on embryo development for ethanol or alcohol can influence embryos through diversity pathways. Our results demonstrate that inhibitory effect of ethanol on early embryos development would appear clearly to be concentration depended when concentration of ethanol was over 0.5% in medium, and harmful impact of ethanol on embryos could last during the whole pregnancy period. Supplement of betaine into embryos culture medium might effectively rescue mouse embryos development damaged by ethanol, and reduce ethanol raised level of global DNA methylation in blastocyst cell.

Early studies had investigated that harmful impact of ethanol by using animal model or *in vitro* culture system^14,15^. But it remains unclear that definite relationship between concentration of ethanol and immediate effect on embryo development because physiological concentration of ethanol in oviduct fluid would variously change. In this study, early embryos development is just obviously inhibited by ethanol at 0.5% in medium, and 2.0% to 2.5% ethanol in medium would thoroughly block blastocyst formation. Similarly to early results, 0.1% ethanol (about 17.7 mM) is helpful to blastocyst formation although we don’t know the true effect of this concentration on long term development. Mikami *et al*.’ s study showed that exposure of L929 cells to 12.5 mM ethanol for 26 h was unable to impair cell division and other cellular functions^32^. So, it is reasonable to postulate that embryo cell can tolerate a lower dose of ethanol in that it can exist as one of products in glycometabolism, and ethanol play a key role in regulation of cell division^33^. All these data suggest that ethanol might not damage the early embryo development only under condition of ethanol concentration being lower than 0.5% in early embryo living environment.

Many studies still tried to find out methods of overcoming damage from ROS caused by excessive ethanol uptake. For example, dietary supplementation of folate might mitigate damaged effect of excessive intake of ethanol on fetuses or brain^34,35^. However, intake of folate might result in damage to the nervous system of fetus because excessive folate could cover expected disease sign caused by deficiency of vitamin B12^36^, or influence cognitive and neural functions in the offspring generation^37^. Recently, a study suggests that significant epigenetic modifications would occur when taking a folate supplement beyond the current advice^38^. Dietary supplement of betaine had also been proven to availably reduce the level of ROS in blood and liver to prevent alcohol inducing fatty liver^39^. Our results confirm alike effect of betaine on reducing the level of ROS in ethanol exposed embryos of. Data show that betaine owns a wider range of safety doses because it can be taken in a higher dosage^40^. Our study indicates that betaine in medium don’t take on obvious inhibitory impact till its concentration reaching 500 μg/ml (4.27 mM). Moreover, the major value of betaine also presents on its excellent capability to improve ethanol impaired embryos to develop for a long term till at last E11.5 stage.

Taken together with existed studies, two probably issues could be hypothesized to explain how betaine improves embryo development besides of protection embryo against ethanol produced ROS. The first, betaine can reduce the intracellular stress reaction to hold osmotic pressure of cell. It had been reported that several amino acids could protect early mouse embryos against increased osmolarity^27^, and betaine as an organic osmolyte can protect mouse preimplantation embryos from the deleterious effects of varying concentrations of NaCl in culture medium^41^, but Baltz *et al*.’s work suggests betaine could not be transported into mouse embryo cells by establishing organic osmolyte transporters^42^. Now, many studies focused on betaine transporter and found several systems including SIT1, which is activated in mouse eggs after fertilization and functions until the 2-cell stage^24,25,28^. Moreover, Wang *et al*.’s work shows embryos would be arrested in the late of 2-cell stage when they are stressed by physiological osmolarity in the absence of organic osmolytes^43^. Besides above, being a natural zwitterionic molecule, betaine had been found to serve as a nontoxic and high efficient cryoprotectant for it presented negligible cytotoxicity even after long-term exposure of cells^44^. But, our study seems not to support this hypothesis of betaine protecting embryos against ethanol by regulating osmolarity in that glycine did not function as expected in this study, although it had been proven that glycine could participate in the process of osmolarity regulation in vitrification of mouse GV oocytes^30^. Meanwhile, study shows that glycine also play a key role in cell signaling transduction for that glycine receptor (GlyR) is a susceptible target for low concentrations of ethanol (5–40 mM)^45^. All these suggest that betaine should play a more important role in embryo cells, not only as an osmolarity regulator,

The second, in embryo cells, betaine might play a novel role in modification process of genome DNA not only to provide active methyls as donor of one-carbon unit metabolism to participate in the synthesis of methionine methylation by reacting with homocysteine. It is hard to explain how the global level of genome DNA methylation decreased when together of betaine and ethanol were added into culture medium. Logically, ethanol could impact the methylation of global genome DNA through several pathways, such as changing activity of enzyme involved in the process of DNA methylation^46^, blocking to provide methyls to metabolism of one-carbon unit or interfering other physiological and biochemical process. So, the status of methylation of genome DNA should intricately vary according to the amount of surround ethanol^47,48^. Character of increasing or decreasing level of genome DNA methylation has been reported in many studies on alcohol effect^49,50^. As being a key donor source of one-carbon metabolism, betaine is usually helpful to maintain the higher level of genome DNA methylation. We guess these higher enriched region of DNA methylation sites perhaps be located in non-transcriptional regulation region for a more stable chromosome structure and this profile could be weaken by ethanol. More experiments should be carried out to explore the real cause of ethanol or betaine impacting embryos development, for example, BSP test of whole genome DNA in blastocyst to find the detail of distribution of 5-meC in all chromosome, which functions in regulating development related genes.

In addition, it has been learned that mouse preimplantation embryos contain endogenous betaine and expression of betaine homocysteine methyltransferase (BHMT) is abundant only at the blastocyst stage^51^. Knockdown of BHMT protein will decrease blastocyst formation and number of cells in ICM, and increase fetal resorption following embryo transfer. Taken together with the published papers, these data suggest that betaine play important roles in regulation of embryos development as a necessary signaling molecular ^51,52^, not only in the cycle of one-carbon metabolism. In this study, the findings that betaine rescued ethanol impaired embryos to undergo a successful journey of development imply a key role of betaine in sustaining genome DNA methylation during the whole gestation period. Thus, we postulate that a higher global level of genome DNA methylation caused by addition of betaine into medium would not hamper normal embryo development.

Totally, our work substantially extends our knowledge of effect of betaine on embryo development, despite the profile of birth and postnatal development of test group and control group had not been analyzed. Even more recently, Karunamuni *et al*. showed that low concentration betaine could alleviate cardiac defects associated with prenatal alcohol exposure^53^. But, more experimental evidences are needed for the safety usage of betaine in clinical treatment and assisted reproductive technology (ART) because a recent study indicates that maternal supplement of betaine during pregnancy and lactation gives rise to different impacts on growth of F1 and F2 offspring in rat liver^54^. Our findings that the ability of betaine to improve development of ethanol treated embryos and the possible pathway of betaine to rescue the global level of genome DNA methylation illustrates that betaine could be used as a physiological agent to handle events associated to excessive uptake of ethanol in reproductive aged woman and human caring of ART, furthermore, betaine might be chosen to reduce ROS level in oocytes and embryos derived from patients with diabetes or others.

## Methods

Unless otherwise stated, all chemicals used in this study were purchased from Sigma chemicals (St. Louis, MO, USA).

### Animals

Virgin 6- to 8-wk-old ICR male female mice, and Kunming vasectomized males were bought from the Experimental Animal Center of Anhui Medical University and were housed in cages maintained under a constant 12-h light/12-h dark cycle at 21–23 °C with free access to standard chow and tap water. The present study was approved by the Animal Care and Use Committee of Fuyang Teachers College. All animal experiments were carried out according to the guidelines of the Animal in Teaching and Research of Fuyang Teachers College.

### Experimental design

Firstly, we investigated the dose effect of ethanol in embryo culture medium (KSOM) on embryo development. Then, a half-inhibitory dose of ethanol was defined through finding out what dose can completely inhibit formation of blastocyst on day 4, when mouse eggs were activated was day 0. In following procedure, IVF embryos were assigned to be cultured in the medium, which betaine (sigma B2629–100G) added into or not, containing half-inhibitory dose of ethanol. Glycine (G8790–100G) was used as an analogue of betaine without methyls. All embryos were then processed for subsequently study.

### Preparation of mouse oocytes, *in vitro* fertilization, preimplantation embryo culture and embryo transferring

Female ICR mice were superovulated by serial injection of PMSG and HCG 48 h apart as described previously^11^. MII oocytes were collected from the oviducts at 14–16 h post-HCG into pre-warmed M2 medium supplemented with 4 mg/ml bovine serum albumin (BSA fraction V, Sigma). The cumulus cells were removed by a shortly incubation at 37 °C in hyaluronidase (0.3 mg/ml, Sigma) in M2 + BSA. The MII oocytes were then washed three times in M2 + BSA and transferred to microdrops of M2 + BSA under paraffin oil pending further treatments.

Conventional IVF was conducted using human tubal fluid (HTF) medium. The obtained sperm from the cauda epididymis of adult male mice were placed in a dish for capacitation in an incubator by keeping them in a 37 °C environment under 5% CO2 and 95% humidity for 1–2 h. Then, an about 1/10 of final volume preincubated, capacitated sperm suspension was added into medium contained cumulus-oocyte complexes. Sperm and oocytes were co-cultured for 3–4 h in insemination medium to achive fertilization.

Fertilized eggs (22–23 h after hCG) as determined by the presence of two pronuclei, were then cultured under optimized culture conditions (KSOM medium without AA) and assessed for their developmental efficiency *in vitro*. ICR females of at least 8 weeks of age were mated with vasectomized Kunming males 3 days prior to blastocyst transfer. Female mouse were checked out for a vaginal plug in the morning after mating with ligated male mouse, and they were supposed to be on day 0.5 of pseudopregnancy. Ten Blastocysts were then transferred into one uterus of the pseudopregnant female according to standard procedures. The implantation sites were visualized through intravenous injection of 0.1 ml of 1% Chicago blue dye solution (Sigma, C8679) in saline 24 hours after blastocysts transferring.

### Detection of the level of ROS in early embryo cells

To detect the level of ROS in cells of the embryos cultured in different condition, collected embryos were incubated with 1 mM of DCFH-DA at 37 °C for 20 min, then embryos were rinsed with PBS and observed by an upright Optiphot-2 microscope (Nikon, Tokyo, Japan) equipped with a TCS SP5 confocal system (Leica Microsystems, Germany) excitation wavelengths of 488 nm. For DCHF-DA can be hydrolyzed to produce DCFH by the esterase and accumulated in the cell. The ROS in the cell can oxidize the DCFH to yield the fluorescent DCF, which can be measured by a fluorescence spectrophotometer at 488 nm excitation and 525 nm maximum emission wavelengths. Thus, the intensity of fluorescence is proportional to the level of ROS in cells.

### Confocal microscopy and quantitative analysis to detect DNA methylation by 5-methylcytosine immunodetection in blastocyst

The procedures for 5-methylcytosine immunodetection and followed observations were described previously, with minor modifications^55^. Briefly, the embryos were washed in phosphate-buffered saline (PBS) and fixed in 4% paraformaldehyde and then stored in 4 °C till following procedure to immunostain embryos from different groups on the same time. In all cases, to wash the embryos in PBS before further processing and to premeabilise them with 0.5% Triton X-100 for 30 min and treated with 4 mol/l HCl for 30 min at room temperature. Then the embryos were treated with 100 mmol/l Tris-HCl (pH 8.5) for 20 min. After washes several times, the embryos were blocked in PBS containing 1% bovine serum albumin (1% PBS-BSA) for 1 h at room temperature. The methylated DNA was visualized using a mouse monoclonal antibody against 5-methylcytosine (anti-5mC, NA81, Merck, Germany). Incubation with this antibody was performed at 4 °C overnight (1:75 dilution in 1% PBS-BSA) followed by washes several times with 1% PBS-BSA, then incubation at room temperature with a fluorescein isothiocyanate (FITC)-conjugated goat-anti-mouse secondary antibody for 1 h. After washes in 1% PBS-BSA several times, the chromatin was stained with propidium iodide (PI, 10 μg/ml) at 37 °C for 15 min, followed by washes several times with 1% PBS-BSA. The embryos were mounted on glass slides.

Observations were performed by using an upright Optiphot-2 microscope (Nikon, Tokyo, Japan) equipped with a TCS SP5 confocal system (Leica Microsystems, Germany) with a Nikon Plan ApoX40 oil-immersion objective and excitation wavelengths of 488 and 514 nm. Serial optical sections (Z-series) were collected at 1-μm intervals through the specimens for each wavelength. The collection of each colour channel was performed sequentially. For each experiment, the same smart gain, smart offset, pinhole and zoom parameters were used. For the quantitative measurements of the integrated fluorescence emitted by each nucleus, the merged images corrected for background were used by subtracting the mean intensity of the cytoplasmic area from the whole image. The nuclear intensities were measured by manually outlining all of the nuclei. The total fluorescence intensity emitted by each individual nucleus was measured using Image Pro-Plus Software and the average intensity for each blastomere was calculated using the ratio of the anti-5mC signal to the PI DNA signal.

### Statistical analysis

Here, all results of quantitative data were shown as Mean ± Standard deviation (SD). All data were analysed by one-way analysis of variance (ANOVA) for assessing any significant difference among the different groups. Only probabilities lower than 0.05 were considered significant.

### Data availability statement

The data in this article is available.
